# Statin Use Improves Overall Survival of Patients with Gastric Cancer after Surgery and Adjuvant Chemotherapy in Taiwan: A Nationwide Matched Cohort Study

**DOI:** 10.3390/cancers12082055

**Published:** 2020-07-25

**Authors:** Pei-Rung Yang, Ying-Ying Tsai, Ko-Jung Chen, Yao-Hsu Yang, Wei-Tai Shih

**Affiliations:** 1 Department of Traditional Chinese Medicine, Chang Gung Memorial Hospital, Chia-Yi 61363, Taiwan; u9302702@cmu.edu.tw (P.-R.Y.); millytsai@cgmh.org.tw (Y.-Y.T.); r95841012@ntu.edu.tw (Y.-H.Y.); 2 School of Traditional Chinese Medicine, Chang Gung University, College of Medicine, Taoyuan 33302, Taiwan; 3 Health Information and Epidemiology Laboratory of Chang Gung Memorial Hospital, Chia-Yi 61363, Taiwan; chenkojung@gmail.com; 4 School of Medicine, Chang Gung University, Taoyuan 33302, Taiwan

**Keywords:** statin, gastric cancer, overall survival, National Health Insurance Research Database, Registry of Catastrophic Illness Patients Database

## Abstract

*Background*: Numerous studies have revealed that statins have antitumor effects in vivo and in vitro. However, few studies have explored the relationship between statin use and the mortality of gastric cancer (GC) patients after treatments. This study examines the relationship between statin use and the overall survival (OS) of GC patients after surgery and adjuvant chemotherapy, using data from the nationwide cohort database of Taiwan. *Methods*: All patients newly diagnosed with GC from 1999 to 2008 in Taiwan were identified from the Registry of Catastrophic Illness Patients Database. Through propensity score matching, statin users were matched to statin non-users at a 1:4 ratio. The relationship between statin use and the OS of patients with GC was estimated through Cox regression models. *Results*: The study cohort included 1835 patients with GC who had received therapies during the study period. The death numbers among statin users (defined as those who used more than 28 cumulative defined daily doses (cDDDs)) and statin non-users were 138 and 895, respectively. A dose–response association was noted between statin use and the OS of patients with GC after treatments. The adjusted hazard ratios were 0.62 (95% confidence intervals (CI), 0.50–0.78) and 0.34 (95% CI, 0.26–0.45) for statin users administered 28–167 cDDDs and >168 cDDDs, respectively, compared with no statin use (<28 cDDDs). *Conclusions*: This study highlights that statin use may dose-dependently improve the OS of patients with GC after surgery and adjuvant chemotherapy in Taiwan. Additional studies are required to confirm the efficacy and safety of statin use.

## 1. Introduction

Gastric cancer (GC), the third leading cause of cancer death globally [1] and the seventh leading cause of cancer death in Taiwan in 2018, is one of the most widespread cancers, particularly in East Asia [2]. Although the incidence of GC has been declining, it remains a fatal disease. Recently, there has been an upward trend of GC incidence among young patients, especially in the Chinese population [3], and a less remarkable decline among women than in men [4]. Each year, approximately 4000 people are diagnosed with GC, and the standard incidence rate is 9.5 cases per 100,000 person-years in Taiwan [5].

Approximately 85–90% of GC cases are due to adenocarcinomas [6]. The etiology of GC is related to diet, *Helicobacter pylori* infection, and the environment [7]. According to the American Joint Committee on Cancer (AJCC), GC is staged into four groups based on tumor, node, and metastasis (TNM) classification. The cancer stage determines the treatment protocol and prognosis. GC is difficult to detect in the early stages because the disease usually progresses gradually in the beginning; thus, there is often a delay in the diagnosis [8].

Surgery, chemotherapy, and radiotherapy are the conventional treatments for GC. Surgical resection and chemotherapy are the first-line therapies for GC. Radiotherapy, immunotherapy, target therapy, and hyperthermic intraperitoneal chemotherapy (HIPEC) are often administered as adjuvant therapy in the advanced stage [9]. The treatment of GC stages Ib, II, and III mainly comprises first-line surgery and subsequent adjuvant chemotherapy. Stage Ia with favorable prognosis can be treated simply with surgery without any other treatment, whereas stage IV with very poor prognosis is treated with chemotherapy as a first-line treatment, usually accompanied by other neoadjuvant or adjuvant therapies [10,11]. More than half of patients with GC are at stages Ib, II, and III, and surgery and chemotherapy are the most common treatments in Taiwan [5]. The recurrence rate after GC surgery is 40%, which is reduced to 13% when accompanied by chemotherapy. The five-year survival rate for patients with GC varies based on the cancer stage and population characteristics [12]. It is less than 40% in most countries and 60–69% in Japan and South Korea [13]. Generally, the five-year survival rate after treatment is 55% in Taiwan [5]. Therefore, developing strategies to effectively enhance the survival of patients with GC is critical.

Statins, which can significantly decrease plasma cholesterol levels, are 3-hydroxy-3-methylglutaryl-coenzyme A (HMG-CoA) reductase inhibitors. It has been confirmed that statins can lower mortality and morbidity in cardiovascular diseases, and they are often used to treat hypercholesterolemia [14]. The anticancer mechanisms of statins involve the inhibition of angiogenesis, inflammation, immunomodulation, and others [9]. In GC cell lines, statins can decrease the level of cellular cholesterol [15], suppress genes involved in cell division, and activate apoptosis [16]. Additionally, animal model studies have shown that statins, combined with radiotherapy or chemotherapy, can reduce tumor volumes [17]. However, clinical studies on this topic are limited. Some previous case–control studies have verified that patients who used statins exhibited a reduced risk of GC [15,18]. Few studies have investigated how statin use improves survival and outcomes after GC diagnosis. One study observed a 17% decrease in the cancer-related death in the United Kingdom [19], and another study revealed an 83% decrease in all-cause mortality after six months of statin use in Korea [20].

Studies have assessed the antitumor and chemopreventive role of statins; nevertheless, the effects of statin use on mortality in patients with GC after treatments remain unclear. This nationwide population-based research investigates the relationship between statin use and the overall survival (OS) of patients with GC after surgery and adjuvant chemotherapy in Taiwan.

## 2. Results

**Figure 1:**
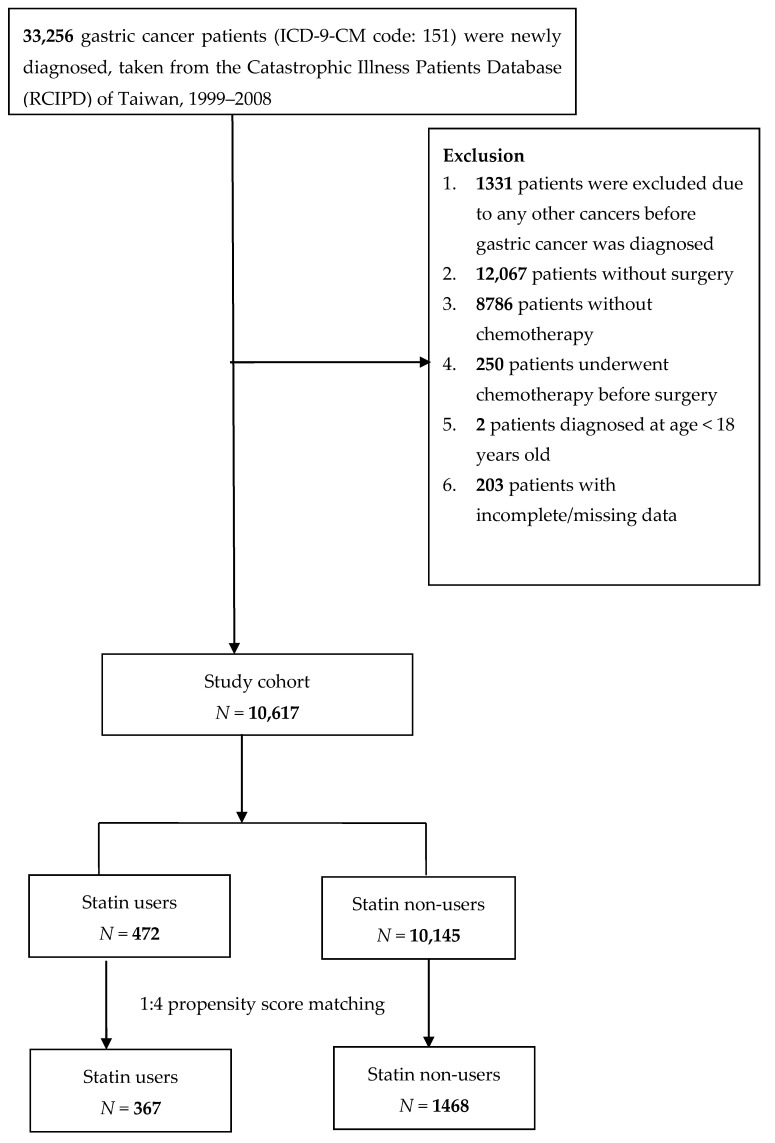
Flowchart of the patient enrollment process of the study cohort and matched cohort.

In this analysis, a total of 10,617 patients with GC were included out of the one million randomly sampled patients. We identified 472 (4.4%) patients who used statins and 10,145 (95.6%) patients who were statin non-users during 1999–2008. After propensity score (PS) matching at a ratio of 1:4, there were 367 and 1468 statin users and non-users, respectively (Figure 1).

The characteristics of the study cohort are summarized in Table 1. The mean age of both statin users and non-users was 64 years. No significant difference was observed between the baseline characteristics of statin users and non-users, except that statin non-users received more triglyceride-lowering drugs. The number of GC patients that died with and without statin use was 138 (37.6%) and 895 (61.0%), respectively.

**Figure 2:**
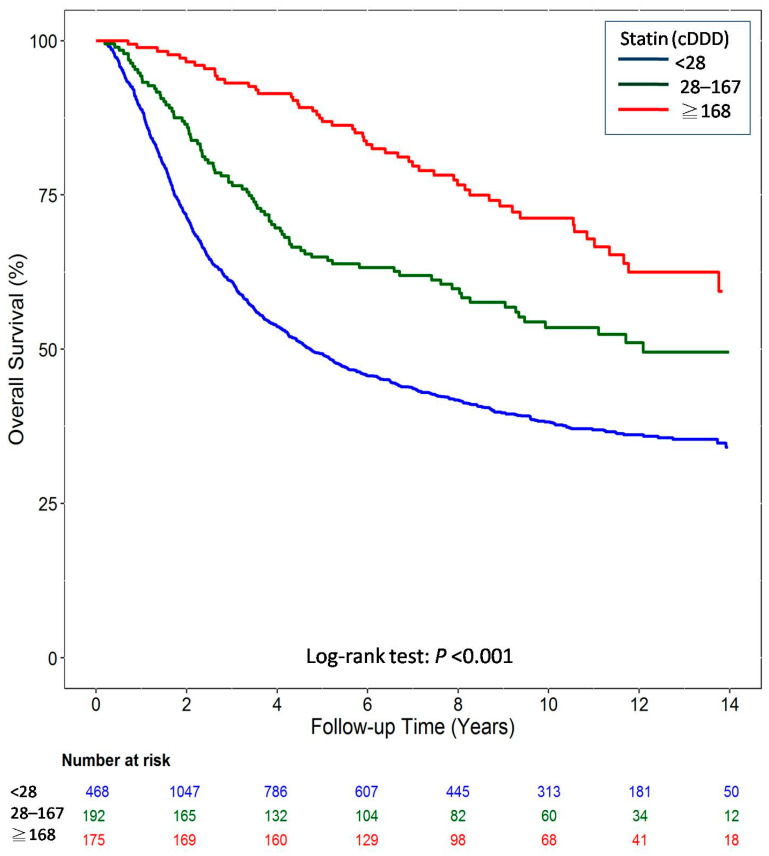
Overall survival of gastric cancer patients after surgery and adjuvant chemotherapy by cumulative defined daily dose (cDDD) of statin use during the follow-up period from the matched cohort.

Figure 2 illustrates the outcomes of the Kaplan–Meier analysis for the matched cohort. The OS improvement showed a progressive dose–response relationship in the matched cohort. The log-rank test revealed a significant increase in the OS (*p* < 0.001, as per the Kaplan–Meier curve) of patients with GC.

Table 2 indicates the dose–response relationship between statin use and the OS of patients with GC after surgery and adjuvant chemotherapy. The adjusted hazard ratios (HRs) were 0.62 (95% confidence intervals (CI), 0.50–0.78) and 0.34 (95% CI, 0.26–0.45) for GC patients with a statin use of 28–167 cumulative defined daily doses (cDDDs) and >168 cDDDs, respectively. Sensitivity analysis showed that statin use had a small effect on the OS of patients with GC in different models with related comorbidities, chemotherapy regimen, and other medicines. This outcome was found to be consistent after excluding some factors causing significant heterogeneity. Sensitivity analysis also demonstrated that the dose–response association between statin use and OS remained in different subgroups of gender and age.

## 3. Discussion

To the best of our knowledge, our study is the first broad random nationwide research to examine the dose–response relationship between statin use and the OS of patients with GC after surgery and adjuvant chemotherapy in Taiwan. This study was controlled for the confounding effects of age, gender, urbanization, income, comorbidities, chemotherapy regimen, and other medicines. The study period was 1999–2008, and the follow-up time was until 31 December 2013. Our subpopulation analysis revealed that the use of statins reduced the mortality in these cohorts. The risk of the death for statin users and statin non-users was 37.6% and 61.0%, respectively, during the study period. After controlling for potential confounders, as the cumulative dose of statins increased a significant tendency towards reducing GC mortality was observed.

We identified the cohort from a population-based and high-quality historical computerized database, which included all the GC patients’ demographic and medical information during the study period. Thus, the possibility of selection and recall bias was eliminated. The study also had a substantial cohort size and a long follow-up period. Moreover, to examine whether the outcomes were consistent, sensitivity analyses were performed with stratification to clarify potential confounders. Especially comorbidities and chemotherapy regimen, which may affect the outcome and prognosis of GC [20], were considered important confounders and examined in this study. No significant changes were observed in the HRs of our measured outcomes in different subgroups.

Statins, which mainly prevent the risk of cardiovascular and cerebrovascular diseases and reduce serum cholesterol levels clinically, are inhibitors of HMG-CoA reductase [14]. Various previous in vitro and in vivo studies have confirmed the antitumor effects of statins on GC. Several potential anticancer mechanisms have been investigated. The basic mechanism of the anticancer effect of statins involves the rate-limiting enzyme in mevalonate synthesis and the inhibition of HMG-CoA reductase, leading to decreased activity of the RAS protein involved in cellular proliferation, differentiation, angiogenesis, and anti-apoptosis [21,22]. An experimental study showed that the use of simvastatin decreased cholesterol levels in the epithelial cells of the stomach and reduced the translocation and phosphorylation of *H. pylori* cytotoxin-associated gene A (CagA), which is considered to play a main role in GC development [15]. Moreover, Histone deacetylase 2 (HDAC2) was proven to be overexpressed in GC. Recent studies have demonstrated that lovastatin can suppress HDAC2 expression by binding to areas near HDAC2 active sites, and HDAC2 inhibition induces the apoptosis of GC cells. Lovastatin has also been found to inhibit the growth of GC cells in vitro in a dose-dependent manner [23]. The results of our research are consistent with the hypothesis concerning the actions of statins.

The chemopreventive effects of statins on GC have been widely discussed in many countries, especially in East Asian countries such as Japan, Korea, and Taiwan, where the GC incidence rate is the highest. Several studies have shown 30–35% reductions in GC risk with statin use [15,18,22,24]. However, only a few observational and clinical research studies have investigated the survival rate of GC patients using statins. Little evidence of progression-free survival or OS difference between statin users and placebo users was noticed [20,25,26]. Poor prognosis of the study-selected advanced GC patients may be the reason why slight significant differences were observed between the overall survival of statin users and statin non-users. A Korean study that examined all-cause mortality and recurrence-free survival in 241 patients with stage II and III GC undergoing radical gastrectomy has provided some evidence. All-cause mortality (HR 0.17; 95% CI, 0.03–0.88) and recurrence-free survival (HR 0.37; 95% CI, 0.10–1.37) in long-term statin users who had been administered statins for more than 6 months were more positive than in short-term users (<6 months) [20]. Another independent UK cohort study demonstrated decreased cancer-specific mortality (adjusted HR 0.83; 95% CI, 0.74–0.92) in GC patients with statin use [19]. In our study, a notable dose–response relationship was observed between statin use and the OS of patients with GC post surgery and adjuvant chemotherapy. The adjusted HRs were 0.62 (95% CI, 0.50–0.78) and 0.34 (95% CI, 0.26–0.45) for statin users with 28–167 cDDDs and >168 cDDDs, respectively. The difference may have been due to practical issues such as different stages of GC, varied patient populations, uneven research duration, or categories of statin exposure [27]. The sample size and the duration of follow-up affected the outcomes. Nonetheless, the survival benefits of statin use for patients with GC after surgery and adjuvant chemotherapy were confirmed in Taiwan, consistent with the result in Asian and Western populations.

This study also has several limitations. First, statin users might not have fully followed the prescribed dosage; however, improvements in the OS of patients with GC following statin use were observed. Second, National Health Insurance (NHI) does not reimburse over-the-counter statin prescriptions, which might have resulted in an underestimation. However, this influence was minimal because only a small number of patients purchase this medicine by themselves. Third, because only the date of death, but not the cause of death, was recorded by the Registry of Catastrophic Illness Patients Database (RCIPD) of National Health Insurance Research Database (NHIRD), the effects of statins on patients with GC who died of a different cause could not be analyzed. Fourth, some potential confounders, including body mass index, smoking and drinking status, which might be associated with the survival of GC patients, were not included in our database. We used alcoholism, smoking-related disorder, diabetes mellitus, hypertension, chronic kidney disease, and liver cirrhosis as additional covariates in the sensitivity analyses. No obvious confounding effects were found because the estimates did not change significantly. Finally, we could not determine the stage of GC from the NHIRD, but we could speculate the stage of GC patients from their treatment protocol. The clinical practice guideline was made by medical experts, according to Taiwan NHI payment regulations.

## 4. Materials and Methods

### 4.1. Data Source

The current research employed the Taiwan NHIRD, which included the RCIPD. By the end of 2010, nearly 23.7 million Taiwanese people (i.e., 99% of the country’s population) were insured under the NHI Program implemented in 1995 [28]. The RCIPD enrolls every patient affected by catastrophic illness that was confirmed through imaging, laboratory, pathology, and clinical diagnosis by NHI Administration experts. The dataset comprises the medical records and information of patients, such as age, gender, date of birth, medical care facilities and specialties, date of outpatient clinical visits or admission, management, procedures and treatment, prescription drugs (name, dosage, and duration), identification number of transfer, and three major diagnoses according to International Classification of Diseases, Ninth Revision, Clinical Modification (ICD-9-CM) codes. Therefore, the NHIRD provides the best platform for epidemiologic research.

Electronic information or patient identity and organization, was encrypted to protect patient privacy. Thus, the informed consent record of patients were not required in our study. The Ethics Review Board of Chang Gung Memorial Hospital, Chia-Yi Branch, Taiwan (201901743B0C501), approved this study on 9 April 2020.

### 4.2. Study Population

Patients newly diagnosed with GC (ICD-9-CM code 151) from 1999 to 2008 who were older than 18 years from RCIPD of Taiwan comprised the study cohort. Patients with GC who had not undergone surgery, chemotherapy, or chemotherapy before surgery were excluded from our cohort. We tracked the study cohort until 31 December 2013, or the date of death. Patients with any other cancer diagnosed before GC and those with incomplete data were also excluded.

The statin prescriptions for this study cohort were collected from RCIPD from the surgery date of patients newly diagnosed with GC during the study period, to the date of death or the end of follow-up. The total exposed dosage, cDDD [29], was used to compare the sum of dispensed DDD of statin usage with the OS of patients with GC. In the study population, patients treated with more than 28 cDDDs of statins after the date of the GC surgery were defined as statin users, whereas those treated with less than 28 cDDDs were defined as statin non-users. We also classified statin users into two groups (28–67 cDDDs and >167 cDDDs) to observe the dose–effect relationship in comparison with statin non-users.

### 4.3. Study Variables

The demographic characteristics of patients were studied to identify the major variables affecting statin use in this GC cohort. In addition to stratification by gender and age, analyses were stratified by the urban levels of NHI registration location, monthly insurance income, and some clinical comorbidities, which were the variables. Comorbidities included diabetes mellitus (ICD-9-CM codes 249–250), hypertension (ICD-9-CM codes 401–405), alcoholism (ICD-9-CM code 303), smoking-related disorders (ICD-9-CM codes 305.1, 491.2, 492.8, 496, 523.6, and V15.82), chronic kidney disease (ICD-9-CM code 585), and liver cirrhosis (ICD-9-CM codes 571.2, 571.5, and 571.6). Chemotherapy regimens (epirubicin-based, mitomycin-based, and taxanes) related to GC and exposure information of other medicine such as triglyceride-lowering drugs, non-statin lipid-lowering drugs, angiotensin-converting enzyme (ACE) inhibitors, aspirin, and non-steroidal anti-inflammatory drugs (NSAIDs) were collected and identified as potential confounders in this study.

### 4.4. Propensity Score Matching

Propensity score (PS) matching was applied to reduce the confounding effects in the two groups [30]. Thus, based on clinical variables comprising age, gender, level of urbanization, monthly insurance income, comorbidities, chemotherapy regimen, and other medicines mentioned above, we used PS to assess the probability of assigning patients as statin users to examine the effect of statin use. Statin users and statin non-users were matched by using PS at a ratio of 1:4.

### 4.5. Sensitivity Analyses

Sensitivity analysis was applied to assess the consistency between statin use and GC mortality. We performed analysis stratified by groups with and without the use of triglyceride-lowering drugs, non-statin lipid-lowering drugs, ACE inhibitors, aspirin, and NSAIDs; and the diseases of diabetes mellitus, hypertension, alcoholism, smoking-related disorder, chronic kidney disease, and liver cirrhosis. We also performed analysis stratified by gender and age from the date of surgery.

### 4.6. Statistical Analysis

Data analysis of descriptive statistics was performed to compare statin users with statin non-users stratified by patient demographics and comorbidities. We performed Pearson’s chi-square test for categorical variables and *t*-test for continuous variables. The Kaplan–Meier method was used to appraise the accumulative probability of OS for statin users and non-users. We used the log-rank test to compare OS curves between the groups. The HRs with 95% CIs were computed using the Cox proportional hazard model adjusted for age, gender, urban level, monthly insurance income, comorbidities, chemotherapy regimen, and other medicines. A two-tailed *p* < 0.05 indicated a significant difference. Data processing and analysis were conducted using SAS version 9.4 (SAS Institute Inc., Cary, NC, USA).

## 5. Conclusions

Statin use may improve OS and reduce mortality in patients with GC after surgery and adjuvant chemotherapy in a dose-dependent manner. Further research must be conducted to improve the clinical evidence on the efficacy and safety of statin use in patients with GC.

## Figures and Tables

**Table 1:** Demographic characteristics of statin users and statin non-users among gastric cancer patients after surgery and adjuvant chemotherapy in Taiwan during 1999–2008 in the matched cohort.

Variables	Statin Users (*N* = 367)		Statin Non-Users (*N* = 1468)		*p*-Value *
	No.	%	No.	%	
**Gender**					0.5543
Female	159	43.3	611	41.6	
Male	208	56.7	857	58.4	
**Age at Surgery**					0.4388
18–64	172	46.9	655	44.6	
≥65	195	53.1	813	55.4	
**Mean (SD)**	64.3 (10.5)		64.1 (12.6)		0.758
**Insured Salaries (NTD$/month) ^a^**					
0+	70	19.1	277	18.9	
1–15,840	38	10.4	154	10.5	
15,841–25,000	185	50.4	733	49.9	
>25,000	74	20.2	304	20.7	
**Urbanization Level**					0.5232
Very High	106	28.9	395	26.9	
High	170	46.3	737	50.2	
Moderate	62	16.9	241	16.4	
Low	29	7.9	95	6.5	
**Comorbidities**					
Hypertension	214	58.3	878	59.8	0.6009
Diabetes Mellitus	122	33.2	467	31.8	0.5996
Alcoholism	8	2.2	23	1.6	0.4150
Smoking-related disorder	40	10.9	166	11.3	0.8244
Chronic kidney disease	13	3.5	64	4.4	0.4848
Liver cirrhosis	14	3.8	61	4.2	0.7682
**Chemotherapy Regimen**					0.9425
Group 1 (epirubicin-based)	29	7.9	105	7.2	
Group 2 (mitomycin-based)	49	13.4	205	14.0	
Group 3 (taxanes)	2	0.5	10	0.7	
Group 4 (others) ^b^	287	78.2	1148	78.2	
**Medication**					
**Triglyceride-Lowering Drugs**					0.0046
User	19	5.2	35	2.4	
Non-user	348	94.8	1433	97.6	
**Non-Statin Lipid-Lowering Drugs**					0.4756
User	8	2.2	24	1.6	
Non-user	359	97.8	1444	98.4	
**ACE Inhibitors**					0.9579
User	98	26.7	390	26.6	
Non-user	269	73.3	1078	73.4	
**Aspirin**					0.3669
User	123	33.5	529	36.0	
Non-user	244	66.5	939	64.0	
**NSAID**					0.7487
User	291	79.3	1175	80.0	
Non-user	76	20.7	293	20.0	
**Death**	138	37.6	895	61.0	

* Pearson’s chi-square test for categorical variables and *t*-test for continuous variables. ^a^ 1 USD = 32.3 New Taiwan Dollars (NTD) in the year 2008. ^b^ Others: cisplatin, carboplatin, oxaliplatin, 5-FU (fluorouracil), capecitabine, TS-1 (tegafur/gimeracil/oteracil), and tegafur.

**Table 2:** Adjusted hazard ratios (HRs) of overall survival of gastric cancer patients after surgery and adjuvant chemotherapy associated with statin use after surgery during the follow-up period in the matched cohort.

Variables	28–167 cDDD				≥168 cDDD			
	HR	95% CI		*p*-Value	HR	95% CI		*p*-Value
Main Model *	**0.62**	0.50	0.78	<0.0001	0.34	0.26	0.45	<0.0001
**Additional Covariates ^†^**								
Main model + Diabetes mellitus	0.64	0.51	0.80	<0.0001	0.33	0.25	0.44	<0.0001
Main model + Hypertension	0.62	0.50	0.78	<0.0001	0.34	0.26	0.45	<0.0001
Main model + Alcoholism	0.62	0.50	0.78	<0.0001	0.34	0.26	0.45	<0.0001
Main model + Smoking-related disorder	0.62	0.50	0.78	<0.0001	0.34	0.26	0.45	<0.0001
Main model + Chronic renal failure	0.62	0.50	0.78	<0.0001	0.34	0.26	0.45	<0.0001
Main model + Liver cirrhosis	0.62	0.50	0.77	<0.0001	0.34	0.26	0.45	<0.0001
Main model + Chemotherapy regimen	0.63	0.50	0.78	<0.0001	0.33	0.25	0.44	<0.0001
Main model + Triglyceride-lowering drugs	0.62	0.50	0.78	<0.0001	0.35	0.26	0.46	<0.0001
Main model + Non-statin lipid-lowering drugs	0.62	0.50	0.78	<0.0001	0.34	0.26	0.45	<0.0001
Main model + ACE inhibitors	0.61	0.49	0.76	<0.0001	0.34	0.26	0.45	<0.0001
Main model + Aspirin	0.60	0.48	0.75	<0.0001	0.33	0.25	0.44	<0.0001
Main model + NSAID	0.60	0.48	0.75	<0.0001	0.32	0.24	0.43	<0.0001
**Subgroup Effects**								
Sex								
Male	0.76	0.57	1.02	0.0674	0.41	0.30	0.57	<0.0001
Female	0.49	0.35	0.70	<0.0001	0.23	0.14	0.40	<0.0001
**Age at Surgery**								
18–64	0.46	0.31	0.67	<0.0001	0.24	0.14	0.41	<0.0001
≥65	0.75	0.57	0.99	0.0402	0.40	0.29	0.55	<0.0001

* Main model was adjusted for sex, age, urbanization, and income. ^†^ The models were adjusted for covariates in the main model as well as each additional listed covariate.

## Abbreviations

cDDDs	Cumulative defined daily doses
GC	gastric cancer
CI	confidence interval
*H. pylori*	*Helicobacter pylori*
HMG-CoA	3-hydroxy-3-methylglutaryl-coenzyme A
OS	overall survival
NHIRD	National Health Insurance Research Database
RCIPD	Registry of Catastrophic Illness Patients Database
ICD-9-CM	International Classification of Diseases, Ninth Revision, Clinical Modification
ACE	angiotensin-converting enzyme
NSAID	non-steroidal anti-inflammatory drug
PS	propensity score
HR	hazard ratio
Cag A	cytotoxin-associated gene A
HDAC2	histone deacetylase 2
